# Investigation of Trends in the Research on Transferrin Receptor-Mediated Drug Delivery via a Bibliometric and Thematic Analysis

**DOI:** 10.3390/pharmaceutics14122574

**Published:** 2022-11-23

**Authors:** Tarnjot Kaur, Jyoti Upadhyay, Sudeep Pukale, Ashish Mathur, Mohd Nazam Ansari

**Affiliations:** 1 Department of Pharmaceutical Sciences, School of Health Sciences and Technology, University of Petroleum and Energy Studies, Energy Acre Campus Bidholi, Dehradun 248007, India; 2 Lupin Research Park, Nande 412115, India; 3 Centre for Interdisciplinary Research and Innovation (CIDRI), University of Petroleum and Energy Studies, Dehradun 248007, India; 4 Department of Physics, University of Petroleum and Energy Studies, Dehradun 248007, India; 5 Department of Pharmacology & Toxicology, College of Pharmacy, Prince Sattam Bin Abdulaziz University, Al-Kharj 11942, Saudi Arabia

**Keywords:** drug delivery, transferrin receptor, bibliometric analysis, performance analysis, scientific mapping, thematic analysis

## Abstract

This study systematically reviews and characterizes the existing literature on transferrin/transferrin receptor-mediated drug delivery. Transferrin is an iron-binding protein. It can be used as a ligand to deliver various proteins, genes, ions, and drugs to the target site via transferrin receptors for therapeutic or diagnostic purposes via transferrin receptors. This study is based on a cross-sectional bibliometric analysis of 583 papers limited to the subject areas of pharmacology, toxicology, and pharmaceutics as extracted from the Scopus database in mid-September 2022. The data were analyzed, and we carried out a performance analysis and science mapping. There was a significant increase in research from 2018 onward. The countries that contributed the most were the USA and China, and most of the existing research was found to be from single-country publications. Research studies on transferrin/transferrin receptor-mediated drug delivery focus on drug delivery across the blood–brain barrier in the form of nanoparticles. The thematic analysis revealed four themes: transferrin/transferrin receptor-mediated drug delivery to the brain, cancer cells, gene therapy, nanoparticles, and liposomes as drug delivery systems. This study is relevant to academics, practitioners, and decision makers interested in targeted and site-specific drug delivery.

## 1. Introduction

Targeted drug delivery is an essential part of drug delivery systems and is used to enhance the therapeutic index of various therapeutic agents [1]. With the advancements in drug discovery, the requirement of a drug delivery system to deliver the therapeutic agent specifically to the target site is crucial in reducing adverse effects and improving clinical benefits. One such approach is drug, metal, or gene targeting via transferrin receptors [2]. Transferrin receptors have excellent potential for targeting drugs in the brain and cancer cells [3]. Different strategies via which to achieve drug targeting include transferrin–drug conjugates [4], anti-transferrin receptor monoclonal antibodies [5], transferrin-bound liposomes [6], and nanoparticles [7]. In this paper, we discuss transferrin and transferrin receptors in detail and how research trends have evolved over the years regarding transferrin/transferrin receptor-mediated drug delivery.

### 1.1. Transferrin

Transferrin is a monomeric glycoprotein [8]. It is present in human serum at a concentration of 200–300 mg/dL [9] and has a half-life of about 8 days [10]. It is an iron-binding protein but can also bind to various other metals, e.g., zinc [11], aluminum [12], cadmium [13], and gallium [14]. In serum, it can exist in various forms such as non-iron-bound transferrin (apo-transferrin), bound to a single ferric ion, i.e., monoferric, or bound to two ferric ions, i.e., diferric transferrin (holo-transferrin) [9].

Transferrin has a polymeric chain and contains 19 disulfide bonds and three carbohydrate moieties; two are N-linked and one is O-linked. Transferrin molecules have two lobes, the N-lobe, which has 336 amino acids, and the C-lobe, which has 343 amino acids; the two lobes are connected by a short linkage sequence [15]. Thus, transferrin has a total of 679 amino acids, and its molecular weight is around 80 kDa [16]. Each lobe has an α-helix domain and a β-sheet domain. The four amino acids, i.e., one aspartic acid, two tyrosine, and one histidine, present in the N- and C-terminal lobes of transferrin, are the binding sites for Fe^3+^ and many other divalent and trivalent metal ions. Therefore, transferrin can be used as a delivery agent for various beneficial or harmful metal ions [17,18]. The iron ion is stabilized at the binding sites by two oxygen molecules donated by carbonate molecules [15].

The highest transferrin concentration is present in hepatocytes [9]. Other cells where it is found are sertoli [19], oligodendroglia [20], myocytes [15], pneumocytes [18], nephrons [21], parietal cells [22], immune cells [23], and cancer cell lines such as human breast and metastatic melanoma [24]; it is also found in bodily fluids such as plasma [9], lymph [23], amniotic fluid [25], cerebrospinal fluid [26], colostrum, and milk [21].

Transferrin + 2Fe^3+^ ⇌ Transferrin(Fe^3+^)_2_. (1)

Various polymorphic forms of transferrin have been detected in more than 30 species, with three major known isotypes: B, C, and D. The C-allele form is most common, particularly C1, whereas, in southwest Africa, the D allele predominates [15]. Egg white contains ovotransferrin [19], and milk, saliva, tears, white blood cells, and mucus contain lactoferrin [18]. The melanocyte surface contains melanotransferrin [27]. Transferrins are acidic, except for lactoferrin. Lactoferrin has an isoelectric point of 8.7. Diferric transferrin species have isoelectric points of 5.6–5.8 [18].

Transferrin controls iron homeostasis through sequestering, binding, transporting, storing, and utilizing iron [28]. In addition to iron absorption, lactoferrin has a role in inflammatory and immune responses [2]. Lactoferrin and ovotransferrin also have antimicrobial activity [9]. Transferrin plays an important function in the body because it helps in the growth, cytoprotection, and differentiation of proliferative, myotrophic, mitogenic, embryo-morphogenic, angiogenic, and neurotropic cells. Due to its iron-binding properties, transferrin is vital for growth, differentiation, and cytoprotection [15].

### 1.2. Transferrin Receptors

Transferrin receptors are membrane-bound glycoproteins responsible for cellular iron uptake [29]. Transferrin receptors are transmembrane homodimers with two identical subunits, each showing a molecular weight of approximately 85 kDa. Each polymeric unit has 760 amino-acid units [30]. Two disulfide bonds, one at cysteine 89 and another at cysteine 98, bind the two monomer units in the transferrin receptor, forming a homodimer [31]. Each subunit can be divided into three parts: the extracellular C-terminal with 670 amino acids, the intramembrane region with 28 amino acids, and intracellular N-terminal with 61 amino acids [29]. The extracellular domain contains two glycosylation sites; one is N-linked at three asparagine residues, and the other is O-linked at threonine. The normal functioning of transferrin receptors requires glycosylation [30]. The intracellular domain has a site for phosphorylation through activated protein kinase C [21]. Each transferrin receptor monomer has three lobes, giving the transferrin dimer a butterfly structure on the plasma membrane [18]. Binding sites are situated in the extracellular domain. Each subunit can bind one molecule of transferrin. The C-terminal domain of transferrin is essential for binding to the transferrin receptor [29].

Transferrin receptors are involved in the transportation and storage of iron [30]. As every cell has a requirement for iron, transferrin receptors are expressed in all cells except mature erythrocytes. Highly proliferative cells have a high requirement for iron; accordingly, transferrin receptor expression is also high. The cells with the highest densities of transferrin receptors are placental tissues, immature erythrocytes, and rapidly dividing cells [29]. Transferrin receptors are found in the gastrointestinal tract (duodenum, ileum, and colon cells), endocrine pancreatic cells, hepatocytes, and Kupfer cells [23]. Transferrin receptors can be identified in the anterior pituitary, thyroid cells, seminiferous tubules of the testis, kidney cells, and basal epidermis cells [21]. Transferrin receptors are localized in brain capillary endothelial cells. Cancer cells have elevated levels of transferrin receptors [18]. There are many studies reporting the presence of transferrin receptors in different cells. However, a limited number of studies have investigated the relative distribution of transferring receptors.

#### 1.2.1. Transferrin Receptor 1 (TfR 1)

TfR 1 internalizes holo-transferrin via clathrin-mediated endocytosis [32]. At pH 7.4, the transferrin receptor is bound to holo-transferrin but not apo-transferrin [29]. In the endosomes, at an acidic pH, ferric ion becomes dissociated from holo-transferrin and the transferrin receptor complex [30]. Ferric ions are converted into ferrous ions by enzyme metalloreductase, and the divalent metal transporter (DMT1) transports them to the cytosol [9]. Recycling endosomes move the TfR 1–transferrin complex to the cell surface, where apo-transferrin becomes separated from the transferrin receptor, and apo-transferrin is released into the bloodstream [9,18].

The expression of TfR 1 is regulated by the concentration and time duration of the presence of iron [33]. When iron is present at high concentrations for a long time, it decreases the expression of TfR 1 and increases intracellular ferritin and vice versa [18]. The hypoxia response element (HRE) promotes TfR gene expression. In iron deficiency and hypoxia, hypoxia-inducible factor (HIF) expression increases, promoting HRE and TfR expression [9]. The 3′ untranslated site of mRNA has five hairpin-like structures called iron-responsive elements (IREs). Iron-regulatory proteins (IRPs) recognize these sites [32]. There are two types of IRPs: IRP 1 and IRP 2. In iron deprivation conditions, both can bind to IREs. IRP 1 has a dual role, playing a role in RNA binding and acting as an enzyme aconitase, depending on the iron status of the cell [9]. In iron deficiency, IRP 1 binds to IREs of ferritin, present on a 5′ untranslated site, and inhibits the translation of ferritin [21]. However, when IRP 1 binds to IREs of the transferrin receptor, it stabilizes the transferrin receptor’s transcription, upregulates TfR 1, and increases the cellular uptake of iron. In iron-rich conditions, IRP 1 binds to mRNA enzymatically, not at the hairpin loop, resulting in the degradation of TfR 1 mRNA. IRP 2 has an equal affinity to mRNA as IRP 1 and binds to all sites; however, it is not active enzymatically. Separate genes encode both proteins [9,18].

Nitric oxide and hydrogen peroxide levels also regulate IRPs in the cell [34]. When iron causes oxidative stress and generates nitric oxide and hydrogen peroxide, IRP 1 is activated by a post-translation mechanism [29]. IPR 2 is activated by de novo protein synthesis [18]. TfR expression is also regulated by cell proliferation. Markedly proliferating cells show a higher expression level of TfRs than nonproliferating cells [21].

#### 1.2.2. Transferrin Receptor 2 (TfR 2)

TfR 2 has two isoforms: α and β [35]. TfR 2α is highly expressed in erythrocyte precursors and hepatocytes and has a molecular weight of 90 kDa [9]. In the cytoplasm, TfR 2α has a short tail of amino acids (1–80) involved in endocytosis, the transmembrane domain, spanning amino acids 81–104, and the extracellular domain of amino acids (105–801), which consists of the protease-associated domain, and can bind to two ferric ions [36]. Hepatic tetraspanin CD81 can interact with the TfR 2α receptor and cause its degradation [9]. TfR 2β receptors are present ubiquitously but at low concentrations, expressed mainly in the brain, heart, and spleen [37]. TfR 2β is a cytosolic protein with a molecular weight of 60 kDa [36].

Many therapeutic and diagnostic agents can bind to transferrin, and the complex thus formed can be targeted to transferrin receptors present at various sites. As transferrin receptors are highly expressed in cancer cells, they can be utilized as potential targets for the delivery of anticancer agents [18]. In order to target transferrin receptors present in cancer cells, various drugs, proteins, or genes are conjugated with transferrin or transferrin-mimicking peptides. This strategy helps increase selectivity and reduces the toxicity and resistance of anticancer drugs [38]. The viral vectors used for gene delivery can be cytopathic or immunogenic. However, nonviral vectors have low transfection efficiency. Nucleic acids conjugated with polycations and crosslinked with transferrin can be used for the delivery of therapeutic genes to cancer cells [18]. Lu et al. [39] proposed the cationic gene vector-mediated delivery of plasmid DNA for gene therapy of prostate cancer. The delivery of non-lipophilic drugs to the brain is limited due to the presence of tightly packed capillary endothelial cells. Transferrin receptors are highly expressed in the capillary endothelial cells of the blood–brain barrier. Therefore, drugs, proteins, and genes linked to transferrin or transferrin-mimicking peptides can be delivered to the brain using transferrin/transferrin receptors [40]. The abovementioned strategy is useful for the treatment of neurological diseases such as Parkinson’s disease, Alzheimer’s disease, stroke, psychiatric disorders, and brain tumors [41].

Targeted drug delivery is required to reduce treatment-related adverse drug reactions. Transferrin receptors are excellent for delivering therapeutic agents such as drugs, metals, and genes to the target site. This bibliometric analysis provides insights into how transferrin receptor-mediated drug delivery has evolved over the years and highlights the contributors to its evolution. Transferrin receptor-mediated targeted drug delivery is beneficial in cancer, gene therapy, and diseases or disorders related to the brain because transferrin receptors are present in high concentrations in cancer cells and in the blood–brain barrier.

## 2. Materials and Methods

This bibliometric analysis provides a framework via which to investigate the intellectual structure of the literature on a specific topic and also assists in identifying research trends [38,39]. Aparicio et al. [40] stated that bibliometric analysis helps one to explore the existing literature on a scientific topic using scientific methods, and Pinto et al. [41] provided evidence on the relevance of bibliometric studies for the identification of keyword statistics, author-specific analyses, and journals publishing relevant work on a research topic. In this work, we applied a bibliometric analysis to transferrin/transferrin receptor-mediated drug delivery and divided our analysis into two parts. The first part of our analysis focuses on the performance analysis of the existing literature. The second part of our analysis contains science mapping of existing studies related to transferrin/transferrin receptor-mediated drug delivery. The data visualizations shown in this study were obtained using R-tool’s Bibliometrix package [42].

**Figure 1:**
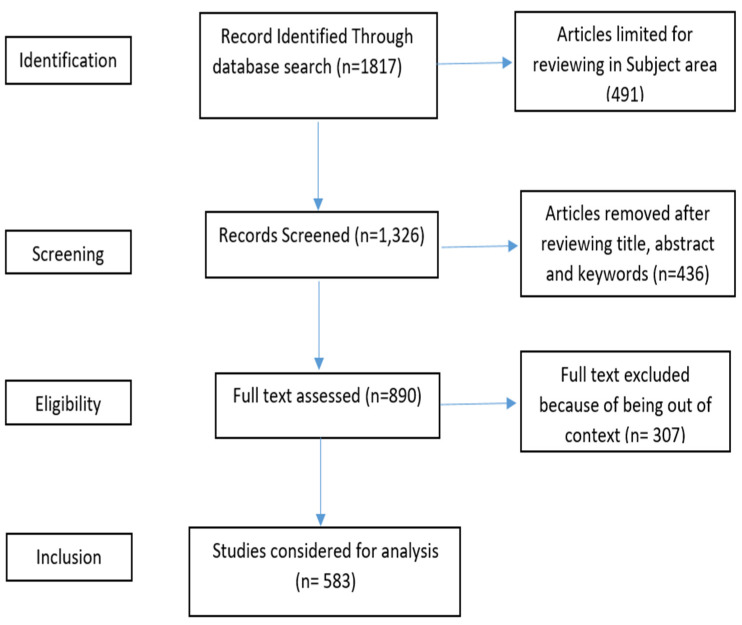
PRISMA flow.

Following existing studies [39,43], we analyzed the existing research on transferrin/transferrin receptor-mediated drug delivery using a bibliometric analysis. The query “TITLE-ABS-KEY (transferrin AND transferrin AND receptor AND “drug AND delivery”) AND (LIMIT-TO (SUBJAREA, “PHAR”) was run in the Scopus database in September 2022, and 583 documents from a total of 1817 documents were retrieved and analyzed. The selected articles for analysis were from 1986 to 2022. All the articles written in English and published in peer-reviewed journals were chosen. The Scopus database was set for broader literature coverage [44]. The sources of the selected studies included studies from pharmacology, toxicology, and pharmaceutics. Figure 1 depicts the details of the selection of the final 583 studies for the bibliometric analysis. Table 1 displays the summary of the key research terms and results from the Scopus database, and this information was further used for the analysis presented in this study. The next section presents the findings from the performance analysis and scientific mapping of the existing literature on transferrin/transferrin-receptor mediated drug delivery.

## 3. Results and Discussion

Transferrin receptors are an essential part of the targeted drug delivery system. They are present at high concentrations in highly proliferating cells, such as erythrocytes, cancer cells, and the blood–brain barrier. Hence, transferrin receptors can be used to target the enhancement of the concentration of a drug in these cells. Transferrin receptors can target various kinds of cancer, neuronal diseases, and the oral delivery of different therapeutic agents. They can also be targeted for gene therapy. The high number of scientific publications is evidence of their importance in targeted drug delivery systems. Several studies in the literature have explored topics such as fragment-based drug discovery [45], pharmaceutical publications [46], ocular drug delivery [47], and progress in pharmaceutical sciences [48] using bibliometric analyses. This bibliometric analysis of transferrin/transferrin receptor-mediated drug delivery provides insights into research trends regarding this topic over the years.

### 3.1. Performance Analysis

**Figure 2:**
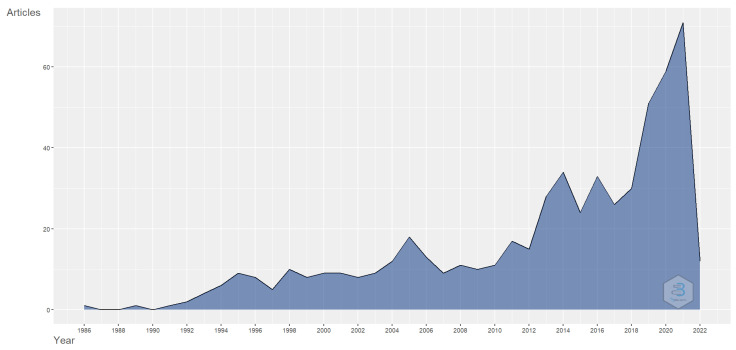
Annual scientific publications relating to transferrin/transferrin receptor-mediated drug delivery. Source: Authors’ elaboration using the Bibliometrix R-package.

Figure 2 shows the number of papers published each year. Research on transferrin/transferrin receptor-mediated drug delivery began in 1986 and continued with an increasing trend toward 2022. A reason for this may be the increase in the understanding of the importance of a novel drug delivery system in site-specific and targeted drug delivery systems with various therapeutic agents and the potential role of transferrin receptors in such a scenario. The research on transferrin/transferrin receptor-mediated drug delivery also gained sudden momentum in 2018.

**Figure 3:**
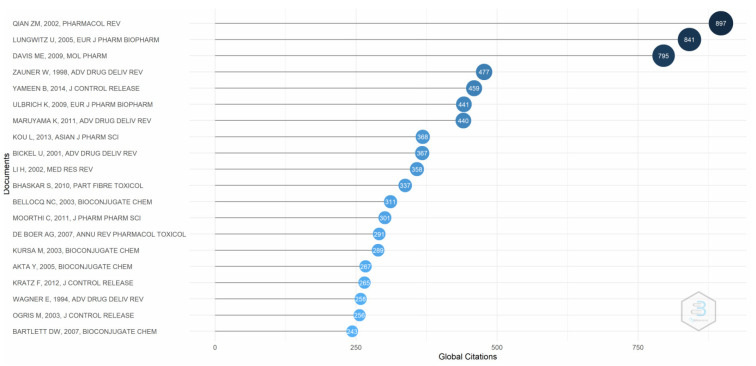
Global citations. Source: Authors’ elaboration using the Bibliometrix R-package.

Figure 3 provides evidence of the global citations for the research published on transferrin receptor-mediated drug delivery. The most cited paper is that by Li and Qian (2002), who examined the existing literature on transferrin/transferrin receptor-mediated drug delivery [18]. Lungwitz et al. [49], who looked at a nonviral gene delivery system based on polyethylene, is the second most cited author. Davis [50], who explored siRNA for targeted drug delivery in humans, is also among the most cited authors. The difference between the number of citations of the top three authors and the remaining authors is visible.

**Figure 4:**
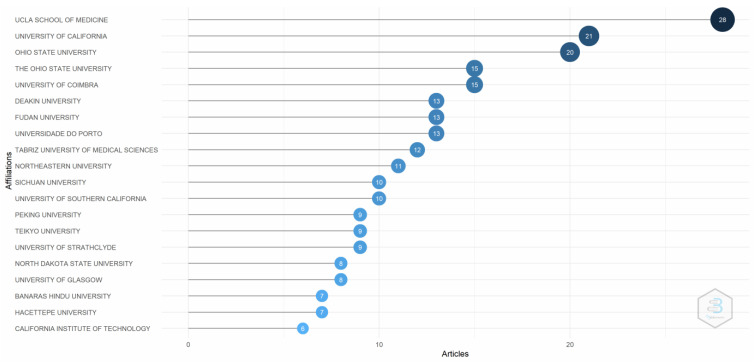
Most relevant affiliations. Source: Authors’ elaboration using the Bibliometrix R-package.

Figure 4 depicts the evidence of the most relevant affiliations. The University of California, Los Angles (UCLA) School of Medicine, contributed the most to publications regarding transferrin/transferrin receptor-mediated drug delivery, followed by the University of California and OHIO State University. Research from emerging market institutions is still limited.

**Figure 5:**
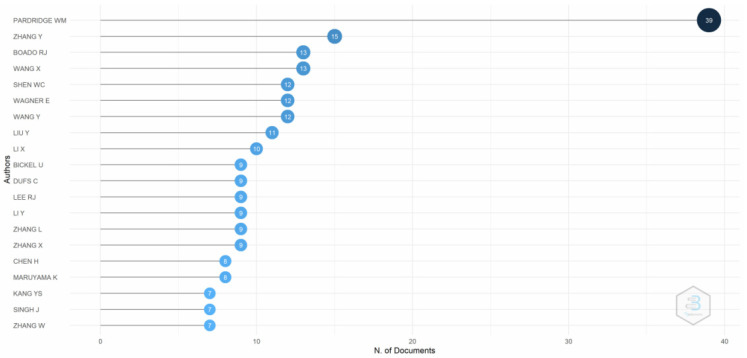
Most relevant authors. Source: Authors’ elaboration using the Bibliometrix R-package.

Figure 5 depicts the evidence of the most relevant authors. Pardridge, who examined the use of transferrin receptors in the human blood–brain barrier to facilitate drug delivery to the brain, is the most relevant author in the literature. Pardridge characterized transferrin receptors in the human brain using monoclonal antibodies in 1987. Pardridge selectively transported antitransferrin receptor antibodies to the blood–brain barrier in rats in 1991. Furthermore, Pardridge, Zhang, and Boado also contributed vitally to the research on transferrin receptor-mediated drug delivery.

**Figure 6:**
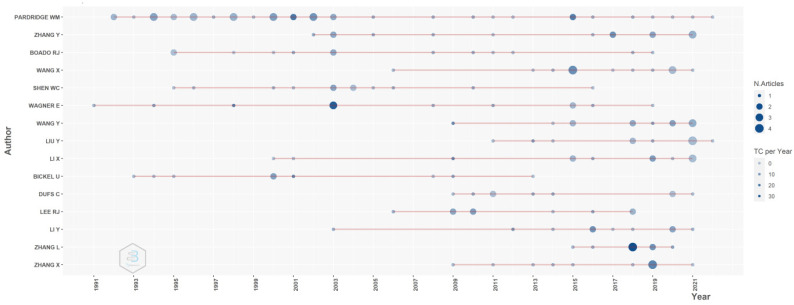
Selected author-specific publications. Source: Authors’ elaboration using the Bibliometrix R-package.

Figure 6 represents the evidence of the selected author-specific publications. It is clear from the evidence that Pardridge and Wagner are the most active researchers in the field of transferrin/transferrin receptor-mediated drug delivery to targeted sites. Pardridge worked on delivering various therapeutic agents to the brain using transferrin/transferrin receptors. He has been the most active researcher from 1992 until present, and he has contributed more than 40 papers. Wagner is one of the earlier researchers who used transferrin/transferrin receptors to deliver drugs or genetic agents for cancer treatment. Zhang L published the maximum number of articles in 2018 and has an interest in a vast area. He explored potential transferrin/transferrin receptor-mediated drug delivery systems for various conditions such as neurodegenerative diseases, cancer, and the oral delivery of peptides for type 2 diabetes mellitus.

**Figure 7:**
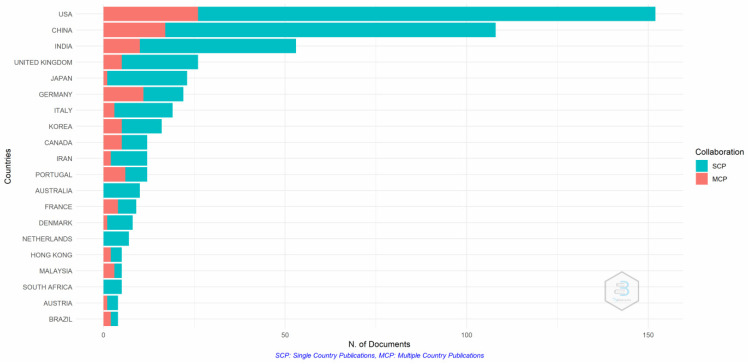
Authors’ countries. Source: Authors’ elaboration using the Bibliometrix R-package.

Figure 7 show that the USA and China have the most active researchers for single- and multiple-country publications. The majority of the papers are single-country publications, which confirms the scientific production. Collaboration between advanced and developing nations is still lacking, as is evident from the single-country publications from South Africa, Australia, and the Netherlands.

**Figure 8:**
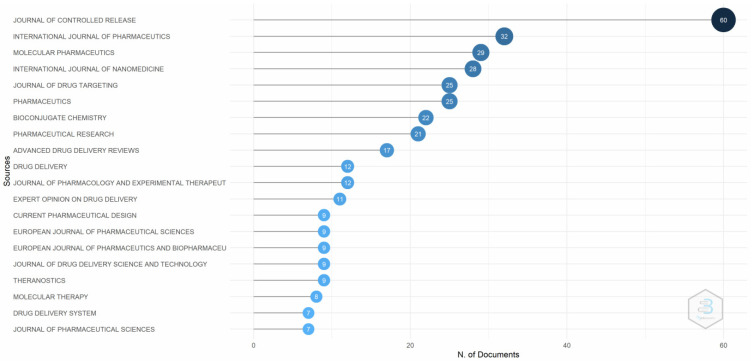
Most relevant sources. Source: Authors’ elaboration using the Bibliometrix R-package.

Figure 8 depicts that the *Journal of Controlled Release* is the most comprehensive source for the targeted delivery of drugs using transferrin receptors. The *Journal of Controlled Release* was established in 1984 and publishes work on drug delivery systems, release mechanisms, and recent developments in pharmaceutics diagnostics. The difference is visible in closely related journals such as the *International Journal of Pharmaceutics*, *Molecular Pharmaceutics*, and the *International Journal of Nanomedicine*.

### 3.2. Science Mapping

#### 3.2.1. Co-Citation Analysis

**Figure 9:**
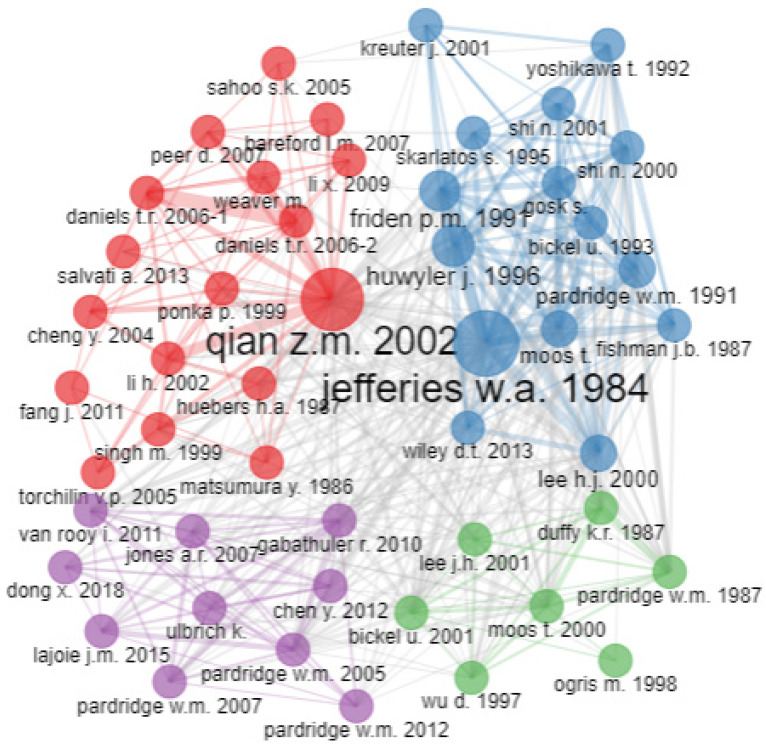
Co-citation network. Source: Authors’ elaboration using the Bibliometrix R-package.

Figure 9 shows four clusters of research co-citations regarding transferrin/transferrin receptor-mediated drug delivery. The size of the knot is related to the number of other authors whom the author collaborated with. A small-sized knot means less collaboration, and a larger-sized knot means more collaboration. A prominent author’s name is a reflection of the author’s contribution. The cluster of red nodes is the largest. Qian reviewed targeted drug delivery systems via transferrin receptors for various drugs, proteins, and genes, as well as the endocytic pathway involved in the cellular uptake of these therapeutic agents. Qian explored the expression of transferrin receptors in different cells under different conditions and the mechanisms involved in the uptake of different agents by transferrin receptors. Researchers also mentioned the potential of targeted drug delivery systems with the aid of transferrin receptors to target the blood–brain barrier. The purple cluster represents targeted delivery to the brain using transferrin receptors. Pardridge reported on the potential use of drug targeting for the brain using transferrin receptors and monoclonal antibodies. Blue and green clusters primarily include earlier studies when transferrin-mediated drug delivery was in its initial phase. Different diseases and delivery systems were being tested for targeted drug delivery via transferrin receptors. Jefferies was among the initial researchers exploring the presence of transferrin receptors in the blood–brain barrier.

**Figure 10:**
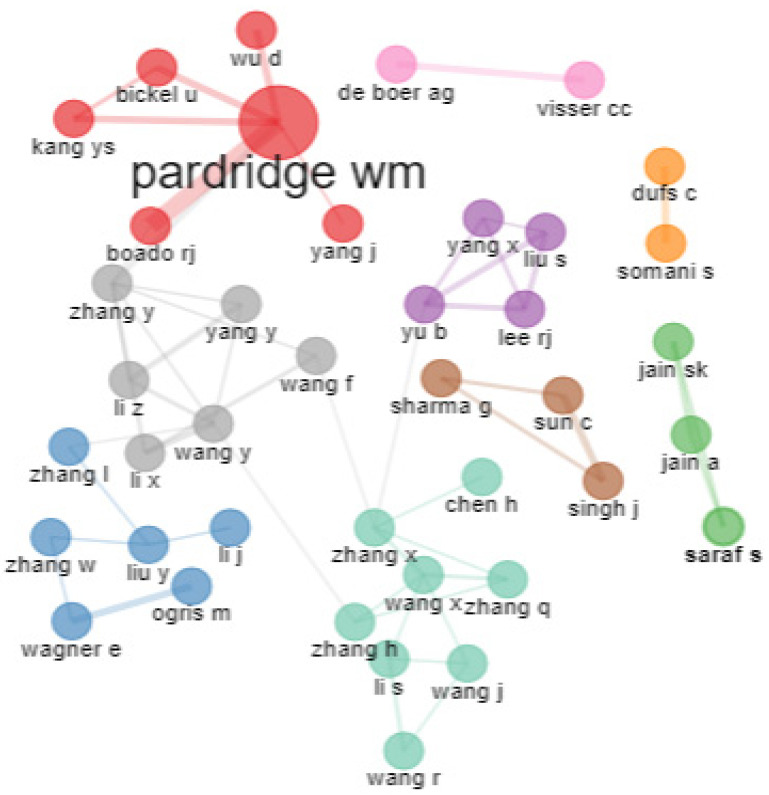
Collaboration network. Source: Authors’ elaboration using the Bibliometrix R-package.

From Figure 10, it is evident that there are nine clusters of collaboration for transferrin/transferrin receptor-mediated drug delivery research. The sea-green color shows the maximum number of associations. The red, gray, and blue clusters show six authors in each group. Pardridge has the highest number of collaborations. Pardridge and Boado RJ investigated drug delivery to the brain using monoclonal antibodies for transferrin receptors and the pharmacokinetic profiles of different agents for brain targeting. In the blue cluster, Wanger and Orgis worked in collaboration on cancer-targeting genes achieved by complexing polyethylene glycol, plasmid DNA, and transferrin to transferrin receptors. The pink and orange clusters show only two authors’ collaboration.

#### 3.2.2. Bibliographic Coupling

**Figure 11:**
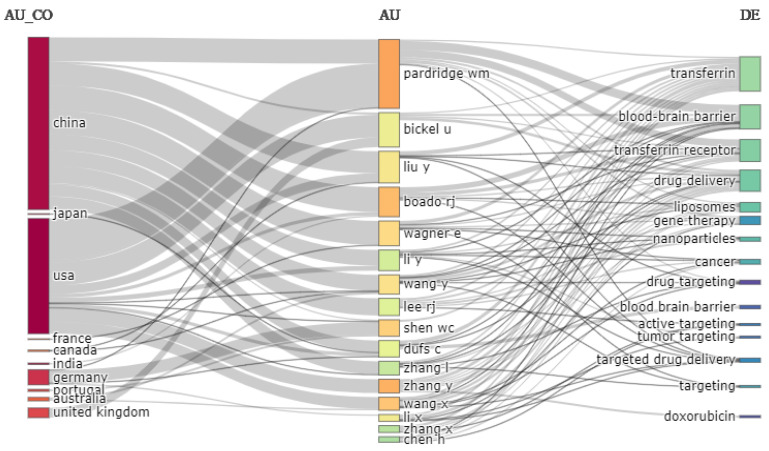
Three-field plot. Source: Authors’ elaboration using the Bibliometrix R-package.

The three field plots in Figure 11 show the author’s country, author, and keywords. Most of the authors are from China, and they studied transferrin receptor-targeted drug delivery to the brain. The country ranked second in the contribution is the USA. Germany, the United Kingdom, and Australia also contributed, whereas the contributions of France, Canada, India, and Portugal were limited. Most studies concern the use of the drug in targeting the brain. Other popular research focuses are cancer and gene therapy. In order to achieve a high concentration of therapeutic agents at the target site, the most employed drug delivery systems are liposomes and nanoparticles.

**Figure 12:**
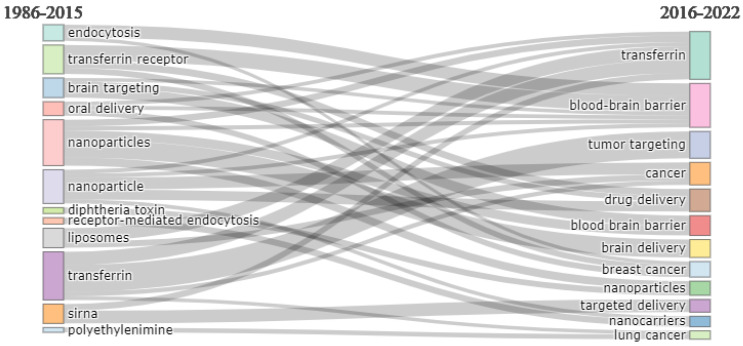
Thematic evolution. Source: Authors’ elaboration using the Bibliometrix R-package.

Figure 12 presents the thematic evaluation of the research on transferrin receptor-assisted drug delivery. It shows that earlier research focused on the endocytosis pathway for transferrin-mediated drug delivery, oral delivery, brain targeting, nanoparticles, liposomes, and siRNA. More recent research involves the blood–brain barrier, cancer, nanoparticles, and nanocarriers.

**Figure 13:**
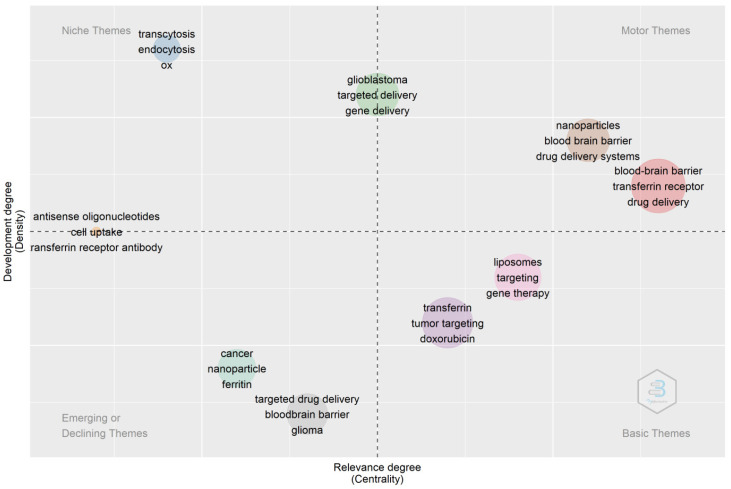
Thematic map. Source: Authors’ elaboration using the Bibliometrix R-package.

The thematic map in Figure 13 depicts that the earlier research focus was on increasing the bioavailability of therapeutic agents via the oral route. Researchers found that cellular uptake via transferrin receptors takes place via endocytic pathways. They explored the transferrin receptor-mediated drug uptake for brain targeting and gene therapy. The drug delivery systems employed were liposomes and nanoparticles. However, more recent research focuses on delivering therapeutic agents across the blood–brain barrier and cancer cells using nanotechnology. Therefore, from the thematic map, it can be concluded that these mechanisms are well understood and well accepted for drug absorption using transferrin receptors. The current focus of research regarding transferrin receptors is brain targeting of the drug for various diseases or disorders of the brain via the use of nanoparticles.

### 3.3. Word and Co-Word Analysis

**Figure 14:**
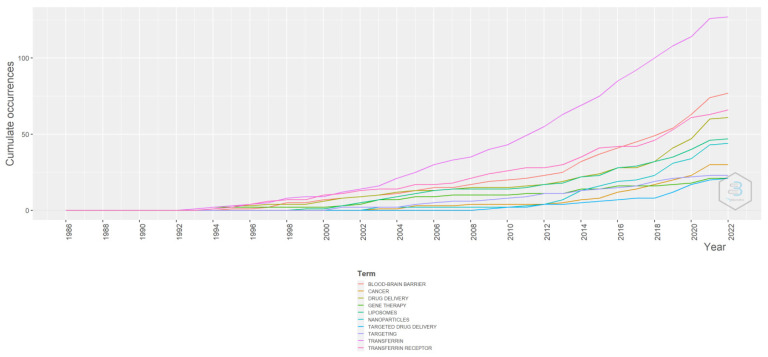
Word growth. Source: Authors’ elaboration using the Bibliometrix R-package.

From Figure 14, it is evident that the use of the word transferrin has increased over the years. It increased at a very high rate after 2014. Similarly, transferrin receptors, cancer, and gene therapy have all been linked, albeit not as frequently as transferrin. The use of liposome formulations has slightly increased, more so than nanoparticles. Targeted drug delivery grew at a languid pace.

**Figure 15:**
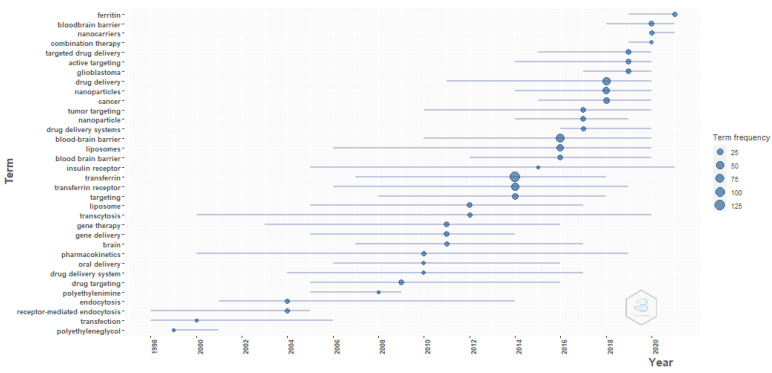
Trending topics. Source: Authors’ elaboration using the Bibliometrix R-package.

Figure 15 show that the blood–brain barrier, glioblastoma, and cancer are trending topics for transferrin/transferrin receptor-mediated drug delivery. The trending formulations are liposomes and nanoparticles. Gene therapy and gene delivery are among other popular subjects. This evidence supports the abovementioned evidence from the thematic maps and word maps. 

**Figure 16:**
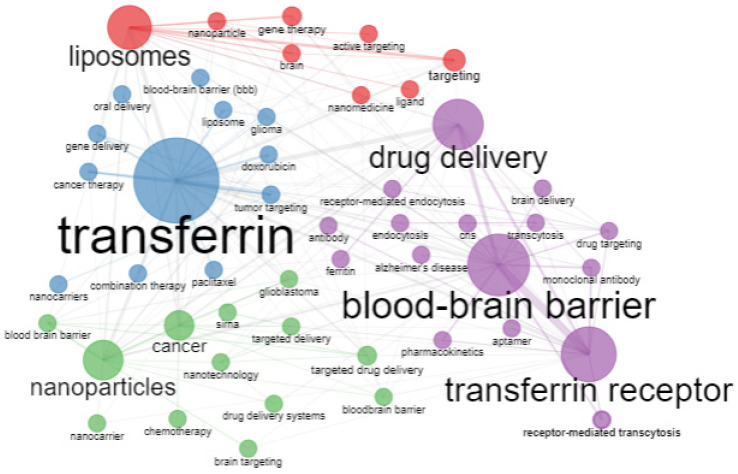
Co-occurrence network. Source: Authors’ elaboration using the Bibliometrix R-package.

Figure 16 shows the co-occurrence network in four clusters. The largest cluster is the purple node; it presents transferrin receptors, the blood–brain barrier, and drug delivery and is in contact with all the other sets. The blue node shows that transferrin is connected with gene therapy, cancer therapy, the blood–brain barrier, glioma, and oral delivery of drugs. A similar finding is valid for the green and red clusters. In the red cluster, liposomes present the most significant node, while, in the green cluster, nanoparticles form the largest node, and how these nodes are connected with smaller nodes is similar to that described above.

## 4. Thematic Analysis

Four themes from the existing research were identified on the basis of the literature review discussed in the previous sections. This thematic analysis depicts the importance of transferrin/transferrin receptor-mediated drug delivery for challenging targets and drug delivery systems. The treatment of various diseases of the brain is complex due to the presence of the blood–brain barrier. Therefore, theme one explores the potential of transferrin receptors to enhance the concentration of therapeutic agents in the brain; these two analyses concern the nonviral delivery of therapeutic genes. Due to the complex nature of cancer cells, the ability of transferrin receptors to deliver therapeutic agents specifically to cancer is discussed in theme three. Theme four explains nanoparticle- and liposome-based drug delivery systems

### 4.1. Transferrin/Transferrin Receptor-Mediated Drug Delivery to the Brain

Bickel et al. [51] targeted transferrin receptors and insulin receptors using OX26monoclonal antibodies and avidin/biotin linkers to deliver neuropeptides across the blood–brain barrier. Walus et al. [52] successfully enhanced the uptake of recombinant human soluble CD4 (rsCD4), a potential anti-HIV agent, by fivefold using anti-transferrin receptor antibodies in primates. Friden et al. [53] reported that radiolabeled recombinant human anti-transferrin receptor fragments are selectively distributed in the brain, but not in any other organ. Therefore, this could be helpful for the delivery of therapeutic agents to the brain. Song et al. [54] increased the brain uptake and decreased the systemic dose of basic biotinylated fibroblast growth factor (bFGF) by conjugating OX26 monoclonal antibodies with streptavidin (bio-bFGF/OX26-SA). Following intravenous injection, they observed significant neuroprotection in the middle cerebral artery occlusion (MCAO) in a rat model. Pardridge et al. [55] studied the effect of human transferrin receptor monoclonal antibodies (hTfRMab) in rhesus monkeys. They found that, to obtain a therapeutic effect, a high dose (25–50 mg/kg) of the low-affinity of TfRMab is required in monkeys, but a low dose (1 mg/kg) of high-affinity TfRMab is sufficient in mice. Plasma clearance follows nonlinear pharmacokinetics, i.e., plasma clearance increases 20-fold with chronic treatment at a low dose (3 mg/kg).

### 4.2. Transferrin/Transferrin Receptor-Mediated Gene Delivery

Viruses such as adenoviruses and retroviruses used for gene delivery are effective ex vivo only, and DNA complexed with cationic liposomes is susceptible to degradation by endonucleases present in the circulation. Shi et al. [56] packaged plasmid DNA in the interior of liposomes stabilized with polyethylene glycol (PEG). In order to target the transferrin receptor, the tip of the PEG stand was conjugated with anti-transferrin receptor monoclonal antibodies. Thus, targeted delivery of β-galactosidase to the brain and peripheral tissues such as the liver and spleen was achieved. In a similar study, Zhang et al. [57] used plasmid DNA encoding antisense mRNA against epidermal growth factor receptors (EGFRs) encapsulated within polyethylene glycol-modified immunoliposomes and targeted transferrin receptors for the treatment of brain cancer. The gene was delivered by weekly intravenous injections in mice. Koppu et al. [58] prepared an intravenous formulation of transferrin-conjugated third-generation diaminobutyric polypropylenimine dendrimers (DABs) complexed with tumor necrosis factor α (TNFα) expressing plasmids derived from the tumor-specific promoter for the treatment of cancer in mice. The gene was mainly expressed in tumors, and treatment was well tolerated. Rodrigues et al. [59] explored the effect of gene delivery systems in mouse brains after intravenous administration. They compared the dual functionalized liposomes of transferrin with three different cell-penetrating peptides (CPPs) (melittin, penetration accelerating sequence-R8, and Kaposi fibroblast growth factor) for their cell internalization and transfection efficiency.

### 4.3. Transferrin/Transferrin Receptor-Mediated Drug Delivery to Cancer Cells

A new approach to minimizing the toxic effects of anticancer drugs limits therapeutic agents’ delivery to cancer cells. Lim et al. reported crosslinking of transferrin receptors with transferrin oligomers, studied endocytosis in cultured tumor cell lines, and found increased intracellular drug concentrations of anticancer drug methotrexate [60]. Nie et al. explored the dual targeting of the gene to integrin and transferrin receptors. They condensed plasmid DNA with polyethyleneimine (PEI); thus, polyplexes were further shielded by PEGylated conjugates. In order to obtain dual targeting, the B6 peptide was targeted to the transferrin receptors, and arginine–glycine–aspartic acid (RGD) was targeted to the integrin receptors [61]. Ge et al. reported dual targeting, the magnetic response through photodynamic therapy with 5-aminolevulinic acid, and transferrin receptor-mediated targeting of the anticancer drug paclitaxel. Paclitaxel entrapped in the lipid compartment of the nanoparticles and 5-aminolevulinic acid along with iron oxide nanoparticles co-encapsulated in the aqueous compartment of the nanocarrier and embedded with transferrin-conjugated Pluronic P123. Thus, the nanocarrier had a controllable “on/off” switch allowing it to increase drug release [62].

### 4.4. Transferrin/Transferrin Receptor-Mediated Drug Delivery Systems

#### 4.4.1. Liposomes

Liposomes tagged with transferrin, preferably holo-transferrin, and liposomes tagged with anti-transferrin antibodies, called immunoliposomes, are two primary drug delivery systems used to target transferrin receptors [6,63]. The binding of the liposomes with polyethylene glycol (PEGylated) prevents the uptake of liposomes by the reticuloendothelial system (RES) and also increases the liposome’s circulation time [64]. Polyethylene glycol (PEGylated) liposomes coupled with anti-transferrin OX26 monoclonal antibodies reduced the distribution of daunomycin to other tissues such as the heart, kidney, lung, spleen, and liver [65]. PEGylated immunoliposomes in Trojan horse liposomes can be used for the noninvasive and nonviral delivery of genes across the blood–brain barrier [66]. The limited uptake of transferrin-tagged liposomes due to receptor saturation across the blood–brain barrier can be overcome by using bifunctional liposomes containing cell-penetrating peptides, such as poly-l-arginine, which are transferrin receptor-targeted [67]. Anti-transferrin antibody-tagged liposomes are better at achieving suitable drug concentrations in the brain than lactoferrin-tagged liposomes [68]. Transferrin liposomes can be tagged with cell penetration enhancer TAT to increase the delivery of various drugs to the brain [69]. Dual targeting, via tagging the liposomes with transferrin and folic acid, can increase the intratumor delivery of anticancer drugs sevenfold [70]. The transferrin/transferrin receptor-mediated drug delivery systems were further advanced using multifunctional liposomes, i.e., the curcumin–lipid ligand due to its affinity for amyloid and transferrin, and the low-density lipoprotein receptor-targeting ligand for the delivery of theranostics across the blood–brain barrier [71]. In addition to using transferrin or anti-transferrin antibodies, transferrin receptors can also be targeted using transferrin-mimicking peptides such as T12, B6, and T7 [72].

#### 4.4.2. Nanoparticles

Various nanoparticles such as PEG–PLA, PLGA, γ-cyclodextrin, solid lipid nanoparticles, lipid polymeric nanoparticles, dendrimeric nanoparticles, gold nanoparticles, and magnetic nanoparticles can be used for the delivery of various therapeutic or diagnostic agents [62,73,74,75,76,77,78,79]. Nanoparticles can be sterically stabilized with PEG to prevent uptake by the reticuloendothelial system and to reduce early clearance from the body [80]. Nanoparticles can be tagged with transferrin ligands, transferrin-mimicking ligands, small peptides, or anti-transferrin monoclonal antibodies [74,76,81,82]. Further drug targeting has been advanced by the use of dual targeting, such as dual receptor targeting, pH-responsive and transferrin receptor targeting, and transferrin receptor targeting and magnetic resonance [62,78,83].

## 5. Future Research Directions

Transferrin and transferrin receptor-mediated drug delivery systems have potential, and there has been extensive research in this field since the early 1990s; however, the clinical application of these is still lacking. Through the literature review, we identified the following as possible directions for future research:

1. Although there has been an increase in the research on transferrin/transferrin receptor-mediated drug delivery systems, most existing studies were carried out in blood–brain barrier models or mouse or rat animal models. However, chronic treatment with anti-transferrin receptor monoclonal antibodies was found to have a low therapeutic index in Rhesus monkeys [55]. Therefore, preclinical studies in primate models are more suitable for future clinical investigation research.
2. Transferrin receptors are highly expressed in the blood–brain barrier, liver, kidney, heart, spleen, and tumor cells [18]. The drug can also be present in high concentrations in other tissues when administered via the intravenous route. Therefore, histopathological and toxicological studies of peripheral tissues should be included in future research.
3. Transferrin receptors are involved in the cellular uptake of iron, and iron is crucial to many biological functions [19]. Therefore, in future research targeting transferrin receptors for the delivery of therapeutic or diagnostic agents, the change in the fate of iron uptake should be evaluated.

## 6. Conclusions

The bibliometric analysis of the evolution of transferrin/transferrin receptor-mediated drug delivery systems over the course of more than 35 years provides evidence for performance analyses and the scientific mapping of the existing literature. A total of 583 papers were analyzed in the literature review. The number of studies has increased over time. The performance analysis showed that research on transferrin/transferrin receptor-mediated drug delivery started in the early 1990s, but gained momentum from 2005 onward. Existing studies have revealed the potential of transferrin receptors for various diseases and drug delivery systems. The USA and China are significant contributors to the research on transferrin receptor-mediated drug delivery. The most studied topic was found to be targeted drug delivery crossing the blood–brain barrier. Other relevant topics of study were liposomes, nanoparticles, gene therapy, and cancer. Until 2015, the existing studies focused on nanoparticles, liposomes, oral delivery, and brain delivery. After 2015, the focus of the research shifted to the blood–brain barrier, tumor targeting, brain delivery, nanoparticles, and nanocarriers. Current research is being conducted on nanoparticles, blood–brain barrier delivery, and brain delivery; similarly, the currently trending topics were found to be glioma, the blood–brain barrier, and cancer.

There is expected to be an increase in scientific publications regarding transferrin/transferrin-mediated drug delivery due to the increasing research interest of scientists in targeted and site-specific drug delivery systems. This expected increase in scientific publications will be similar to the trend observed over the last several years, especially among the countries listed in this study. This study is expected to increase interest among academics, practitioners, and decision makers in extending the findings of this study in the field of transferrin/transferrin-mediated drug delivery. This study is relevant to academics, practitioners, and decision makers interested in targeted and site-specific drug delivery.

## Figures and Tables

**Table 1:** Data description.

Description	Results
Main information about the data	
Timespan	1986–2022
Sources (journals, books, etc.)	130
Documents	583
Average years from publication	9.23
Average citations per document	50.04
Average citations per year per doc	4.87
References	41,320
Document Types	
Article	398
Book chapter	10
Conference paper	14
Editorial	7
Note	2
Review	145
Short survey	3
Document Contents	
Keywords plus ID	6002
Author’s keywords (DE)	1365
Authors	
Authors	2245
Author appearances	3148
Authors of single-authored documents	26
Authors of multi-authored documents	2219
Author Collaboration	
Single-authored documents	34
Documents per author	0.256
Authors per document	3.91
Co-authors per document	5.48
Collaboration index	4.11

## Data Availability

Not applicable.
